# Light sheet fluorescence microscopy guided MALDI-imaging mass spectrometry of cleared tissue samples

**DOI:** 10.1038/s41598-020-71465-1

**Published:** 2020-09-02

**Authors:** Andreas Blutke, Na Sun, Zhihao Xu, Achim Buck, Luke Harrison, Sonja C. Schriever, Paul T. Pfluger, David Wiles, Thomas Kunzke, Katharina Huber, Jürgen Schlegel, Michaela Aichler, Annette Feuchtinger, Kaspar Matiasek, Stefanie M. Hauck, Axel Walch

**Affiliations:** 1 Research Unit Analytical Pathology, Helmholtz Zentrum München, 8576 Neuherberg, Germany; 2 Research Unit Neurobiology of Diabetes, Helmholtz Zentrum München, 85764 Neuherberg, Germany; 3 Institute for Diabetes and Obesity, Helmholtz Zentrum München, 85764 Neuherberg, Germany; 4 German Center for Diabetes Research (DZD), 85764 Neuherberg, Germany; 5 Division of Metabolic Diseases, Technische Universität München, 80333 Munich, Germany; 6 arivis AG, 80636 Munich, Germany; 7 Institute for Pathology, Department of Neuropathology, Technische Universität München, 80333 Munich, Germany; 8 Institute for Veterinary Pathology at the Centre for Clinical Veterinary Medicine, Ludwig-Maximilians-Universität München, 80539 Munich, Germany; 9 Research Unit for Protein Science, Helmholtz Zentrum München, 85764 Neuherberg, Germany

**Keywords:** Mass spectrometry, Light-sheet microscopy

## Abstract

Light sheet fluorescence microscopy (LSFM) of optically cleared biological samples represents a powerful tool to analyze the 3-dimensional morphology of tissues and organs. Multimodal combinations of LSFM with additional analyses of the identical sample help to limit the consumption of restricted specimen and reduce inter-sample variation. Here, we demonstrate the *proof-of-concept* that LSFM of cleared brain tissue samples can be combined with Matrix Assisted Laser Desorption/Ionization-Mass Spectrometry Imaging (MALDI-MSI) for detection and quantification of proteins. Samples of freshly dissected murine brain and of archived formalin-fixed paraffin-embedded (FFPE) human brain tissue were cleared (3DISCO). Tissue regions of interest were defined by LSFM and excised, (re)-embedded in paraffin, and sectioned. Mouse sections were coated with sinapinic acid matrix. Human brain sections were pre-digested with trypsin and coated with α-cyano-4-hydroxycinnamic acid matrix. Subsequently, sections were subjected to MALDI-time-of-flight (TOF)-MSI in mass ranges between 0.8 to 4 kDa (human tissue sections), or 2.5–25 kDa (mouse tissue sections) with a lateral resolution of 50 µm. Protein- and peptide-identities corresponding to acquired MALDI-MSI spectra were confirmed by parallel liquid chromatography tandem mass spectrometry (LC–MS/MS) analysis. The spatial abundance- and intensity-patterns of established marker proteins detected by MALDI-MSI were also confirmed by immunohistochemistry.

## Introduction

Multimodal combination of 3-dimensional (3D)-imaging methods with additional, tissue-based analysis techniques that can subsequently be performed on the identical sample(s) is advantageous in diverse experimental settings. Combined 3D-imaging- and subsequent advanced analysis approaches allow for a precise definition of the tissue localizations to be further analyzed, and enable a direct morphological co-localization of the results of different downstream analyses types and their integration into superordinate functional concepts. Multimodal combination of different analytical methods for examination of various morphological, functional, or molecular parameters within the identical sample material can contribute to expand the spectrum of findings in a given sample, and efficiently reduce inter-sample-variation, unspecific “background noise”, as well as technical bias^1,2^. Therefore, multimodal analysis approaches are also ideal for cross-validations and confirmation of previous findings. Moreover, they can help to reduce the number of experimental animals sacrificed in a study, and are also preferable, if the amounts of available sample materials are limited.

Light sheet fluorescence microscopy (LSFM) of optically cleared biological tissue samples has rapidly evolved during the last decade and established as a powerful tool for 3-dimensional analysis of tissue morphology applied in various life science disciplines^3–5^. In LSFM, a laser light sheet of adjustable wave-length illuminates a thin (approximately 5 µm thick), orthogonal plane of the examined sample. Fluorescent signals emitted by excited molecules within the plane of illumination are detected, resulting in a 2D cross sectional fluorescence image with a lateral resolution of less than a few microns, depending on the objective used, the magnification, and the wave-length of the fluorescent light^6^. During examination, the light sheet plane is moved along the z axis of the sample^3–5^. A z-stack consisting of serial 2D images is computed to a 3D image of the sample that can be freely rotated, sectioned, and viewed in all directions of space. Compared to e.g., confocal microscopy, image acquisition is much faster with LSFM, yet the optical resolution in the z-axis is lower. To enable a complete penetration of the sample by the laser light sheet, the sample is usually optically cleared, i.e., made transparent by adjusting its optical refractive index to that of the surrounding medium, using different chemical solvents, hydrogel-embedding techniques, or aqueous hyperhydrating techniques. A large number of different methods and protocols, such as CLARITY, PACT, PARS, iDISCO, 3DISCO, uDISCO, BABB, CUBIC, TDE, FRUIT, CLEAR T (reviewed in^4^), vDISCO, and ECi^7,8^ have been developed for tissue clearing, optimized for different sample types and various downstream applications. The fluorescence signals detected by LSFM are either derived from the natural autofluorescence of different structural tissue components in the sample, or they originate from the expression of fluorescent reporter molecules such as e.g., GFP in transgenic experimental animals, or from in vivo administered fluorescent-labeled exogenous substances. Alternatively, distinct target-antigens can be detected by whole-mount immunolabeling of the sample with specific antibodies labeled with fluorescent markers (fluorescence-labeled primary and/or secondary antibodies). Noteworthy, several chemical tissue clearing protocols and subsequent LSFM work reasonably well using formalin-fixed paraffin-embedded (FFPE) specimen, after deparaffinization of the samples^9,10^. LSFM is used for visualization and analysis of complex 3D morphological properties of organs and tissues in physiological conditions, during distinct stages of development, or in states of disease. With steadily advancing computational power and the availability of excellent new software tools for image data analysis, this technique is about to revolutionize the way of analysis of 3D tissue morphology^11^. Next to the 3D visualization of the complex architecture of many tissues, the possibility to directly analyze quantitative parameters of the tissue morphology by LSFM holds the great potential to provide a fast and feasible alternative to the often complex, time-consuming and cumbersome classical quantitative stereological approaches that depend on examination of 2D sections. Multimodal combination of LSFM with additional tissue-based morphological analyses is particularly beneficial, since it enables the integration of the results of different analysis methods into the superordinate 3D morphology of the examined sample. For example, LSFM has been demonstrated to be successfully combinable with histology, and immunohistochemistry (IHC)^9,10,12^.

Here, we asked, if LSFM of optically cleared tissue samples would also be combinable with Matrix Assisted Laser Desorption/Ionization-Mass Spectrometry Imaging (MALDI-MSI). MALDI-MSI is used to detect and analyze the spatial abundance patterns of different molecule classes including small molecule metabolites, lipids, and proteins directly in histological sections of the tissue^13–18^. Briefly, in MALDI-MSI, histological tissue sections are mounted on a conductive microscope slide and coated with homogenous layers of a matrix-solvent mixture. Depending on the class of analyzed target molecules, commonly used matrices include e.g., 2,5-dihydroxybenzoic acid (DHB), alpha-cyano-4-hydroxycinnamic acid (CHCA) or sinapinic acid (SA) for detection of drugs, lipids, peptides or proteins^16,18^. The solvent extracts the analyte molecules from the section followed by matrix/analyte co-crystallization preserving analytes position to their origin in the tissue section beneath.

In the mass spectrometer, the matrix-coated section surface is then scanned in two dimensions by short impulses of focused laser beam spots. The matrix absorbs and transfers the laser energy to the matrix-bound analytes, enabling desorption and ionization. The ablated molecular ions are then accelerated by an electric field in the vacuum of the mass spectrometer (MS), separated, and detected according to their *m/z* ratio^16,18^.

For MSI analyses, various types of MALDI-MSI instruments and a plethora of different analytical techniques and tissue processing protocols are available using diverse matrices, solvents and analysis settings, optimized for analysis of different molecule-classes (*m/z* ranges) in different types of tissue samples^16,19^.

Using suitable software tools, the mass spectra recorded at each single examined location of the section are then analyzed and computed to visualize the spatial distribution, and the relative intensity of each identified analyte, corresponding to the optical image of the section. Depending on the applied method, the analysis type and the examined sample, several thousand different analytes can be detected and analyzed in a single MALDI-MSI experiment^16,18,20^. Thus, MALDI-MSI combines the advantages of histomorphological analyses and liquid-chemistry approaches for identification and quantification of molecular analytes. Following MALDI-MSI, the matrix can be washed off the slide, and the section can be stained with histological dyes, such as hematoxylin and eosin. Furthermore, MALDI-MSI analyses can also be combined with a variety of other imaging modalities, providing additional morphological and/or biochemical information from the identical tissue section, or sample^14,17^. Previously reported multimodal (MALDI)-MSI analysis approaches e.g., include the combination with autofluorescence-microscopy^21,22^, Raman spectroscopy^23^, magnetic resonance imaging (MRI)^24,25^, histology, immunohistochemistry, and laser scanning confocal microscopy^26^. Using appropriate alignment methods^22,24,27^, the images acquired by MALDI-MSI and additional imaging modalities can be exactly overlaid and thus directly be related to e.g., distinct morphological features of the examined tissue, such as different structural compartments, cell types, pathological alterations, etc.

Several examples confirm the usefulness of multiplex MALDI-MSI analyses for detection of spatial metabolic, lipidomic, and proteomic differences in a broad spectrum of tissues in various disease entities, and as a meaningful supplement to histopathological diagnostics^20,28–37^.

Of note, MALDI-MSI principally also works on paraffin-embedded (PE) tissue samples, enabling retrospective analyses of archived PE-sample materials stored in pathology archives and tissue-biobanks^19,38–40^. However, there are some restrictions for MSI of paraffin-embedded specimen, depending on the applied method of tissue fixation, as well as on the class of molecules to be detected by MALDI-MSI. Whereas MALDI-MSI detection of small molecule metabolites is quite unproblematic in formalin-fixed (FF) PE samples, the molecular cross-linking introduced by formalin fixation severely limits the analysis of proteins. For MALDI-MSI of proteins in FFPE sections, additional steps, such as e.g., enzymatic digestion (trypsinization) of the section surface have to be applied, and analysis is based on the detection of the masses of the released trypsin-digestion fragments of the proteins^14,41^. Here, the use of ethanol-preservation, or alternative, formalin-free fixation solutions, such as e.g., PAXGene, or RCL2/CS100, prior to paraffin-embedding is advantageous, allowing for MALDI-MSI of intact proteins, while also maintaining a comparably well preservation of the histomorphology of the tissue^42–44^.

In the present report, we provide the *proof-of-principle*, that differential, high quality MALDI-MSI-spectra of peptides and proteins in mass ranges from 800 Da–2.5 kDa can be acquired from paraffin sections prepared from human and murine brain tissue samples following chemical clearing and examination by LSFM, even, if archived FFPE-tissues are used as initial samples.

Combination of LSFM of cleared tissue samples and subsequent MALDI-MSI represents a technically challenging task that has not been addressed so far. The combinatory LSFM/MALDI-MSI approach demonstrated in the present study utilizes the high instrumentations of both analysis methods, allowing for expeditious, 3D-LSFM guided IMS of defined regions of interest within optically cleared tissue samples.

## Results

### Light sheet fluorescence microscopy (LSFM) allows fast and precise definition of 3D tissue regions of interest in differentially fixed, optically cleared brain samples for subsequent MALDI-MSI analysis

**Figure 1:**
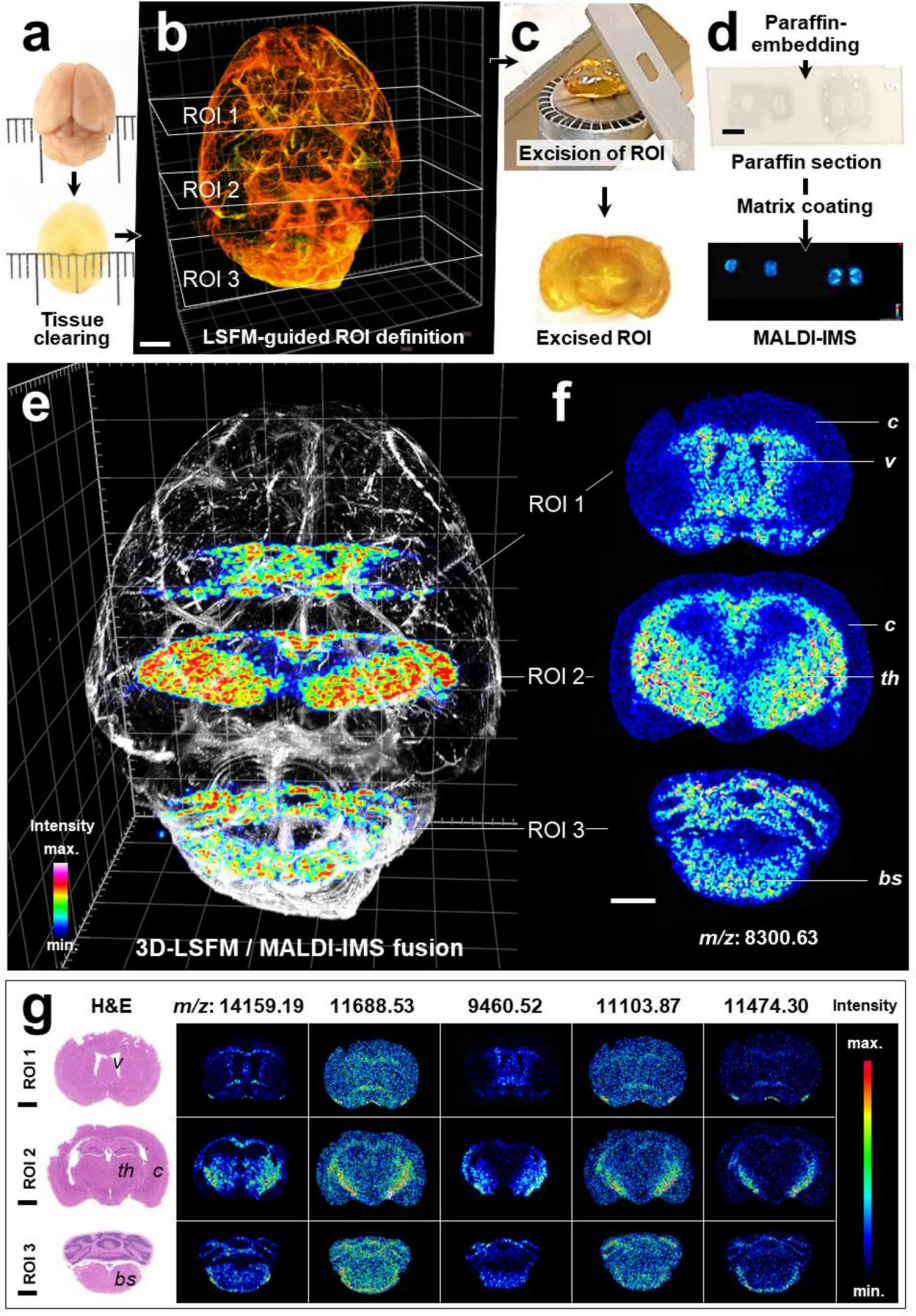
3D-Light sheet fluorescence microscopy (LSFM) guided MALDI-imaging mass spectrometry (IMS) in an optically cleared mouse brain. (**a**–**d**) Sequence of tissue processing steps. (**a**) Optical clearing (3DISCO^45^) of a fluorescent-labelled, PaxGene-fixed mouse brain^46^. (**b**) LSFM-3D reconstruction of the brain. Fluorescent-labelled blood vessels are featured in red-yellow color, the choroid plexus is visualized in green color. Three tissue regions of interest (ROI 1–3) at the level of the cerebral cortex/striatum (ROI 1), the diencephalon (ROI 2), and the cerebellum (ROI 3), are chosen for subsequent MALDI-MSI. Bar = 500 µm. (**c**) Tissue sections corresponding to the LSFM-defined ROIs are excised from the cleared tissue. For this, the use of a sample positioning and sectioning device as shown in Supplemental Fig. 2 can be helpful. In image (**c**), the excised ROI 2 (frontal diencephalic brain section) is shown. Bar = 1 mm. (**d**) The excised tissue is embedded in paraffin. Paraffin sections are coated with MALDI matrix and subsequently subjected to MALDI-MSI. Bars = 5 mm. (**e**–**g**) MALDI-MS images of selected annotated proteins confirmed by LC–MS/MS. (**e**) Fused image of the 3D-LSFM reconstruction of the cleared brain and MALDI-MS images of guanine nucleotide-binding protein subunit gamma-3 (GNG3, *m/z*: 8,300.63), acquired in paraffin sections of the three excised regions of interest (compare to Supplemental video 1). For better visualization, the blood vessels of the brain are shown in grey color. (**f**) MALDI-MS images of GNG3. The spatial distribution of GNG3, as detected by MALDI-MSI, corresponds to the physiological anatomical spatial abundance pattern of the protein in the murine brain^47^. Distinct brain structures are indicated for orientation: Cerebral cortex (*c*), ventricles (*v*), thalamus (*th*), brain stem (*bs*). (**g**) Representative MALDI-MS images of five different putative identified proteins (indicated by their *m/z*) detected in paraffin-sections of ROI 1–3 and corresponding HE-stains prepared after matrix removal: *m/z* 14,159.19 (Short coiled-coil protein, SCOC); *m/z* 11,688.53 (NADH dehydrogenase [ubiquinone] 1 beta subcomplex subunit 3, NDUFB3); *m/z* 9,460.52 (40S ribosomal protein S27, RPS27); *m/z* 11,103.87 (28S ribosomal protein S36, mitochondrial, MRPS36); *m/z* 11,474.30 (60S acidic ribosomal protein P1, RPLP1). Protein identification was confirmed by LC–MS/MS analysis using SwissProt Database. Signal intensity differences are indicated by different colors. Note the distinct spatial patterning of MALDI-MSI signals corresponding to different anatomical tissue compartments of the brain. Bars = 1 mm (**e**–**g**).

To demonstrate the combinability of optical tissue clearing and LSFM with subsequent MALDI-MSI using standard LSFM- and MALDI-MSI instrumentation, routine sample processing protocols, and commonly examined types of tissue specimen, two separate experiments were performed, using murine brain tissue samples (Experiment No. 1), as well as archived sample materials of human brain tissue (Experiment No. 2) from different previous studies. A detailed schematic presentation of the designs and work-flows of Experiment No. 1 and No. 2 are provided in the supplementary data (Experiment No. 1 and No. 2, Supplemental Fig. 1). In Experiment No. 1, a mouse brain fixed with a commercially available formaldehyde-free fixation solution (PaxGene, PreAnalytiX, Switzerland) was optically cleared according to a modified 3DISCO protocol (Fig. 1a) and examined by LSFM^45,46^. The examined brain was derived from a previous study investigating the distribution of leptin, an adipose tissue derived peptide hormone in the brain. The mouse had been i.p.-injected with fluorescent CW800-labeled recombinant mouse leptin, and i.v.-injected with a fluorescent lectin-647 to visualize blood vessels^46^. The 3D morphology of fluorescently labelled blood vessels and the distribution of the injected fluorescence-labeled leptin protein in different anatomical regions of the mouse brain, including the median eminence, the hypothalamus and the choroid plexus, was visualized by LSFM (Fig. 1b). Using LSFM, three representative coronal section planes at the level of the cerebral cortex/striatum, the thalamus, and the cerebellum/brain stem could precisely be defined as ROIs for subsequent MALDI-MSI analysis. These ROIs were excised from the cleared brain (Fig. 1c), using unambiguously identifiable anatomical landmarks. To facilitate cutting of straight, controlled section planes, a self-constructed sample positioning and sectioning device was used (Supplemental Fig. 2). For MALDI-MSI, the excised tissue regions of interest (ROIs) were then embedded in paraffin. Sections of approximately 3 µm thickness were cut from the paraffin block and processed for MALDI-MSI (Fig. 1d). Additional tissue sections were subsequently cut from the same block and processed for proteomic analysis by liquid chromatography-tandem mass spectrometry (LC–MS/MS). After acquisition of MALDI-MSI spectra, the coated matrix was removed from the MALDI-sections, and the identical the sections were subsequently stained with hematoxylin and eosin (H&E) (Fig. 1g).

**Figure 2:**
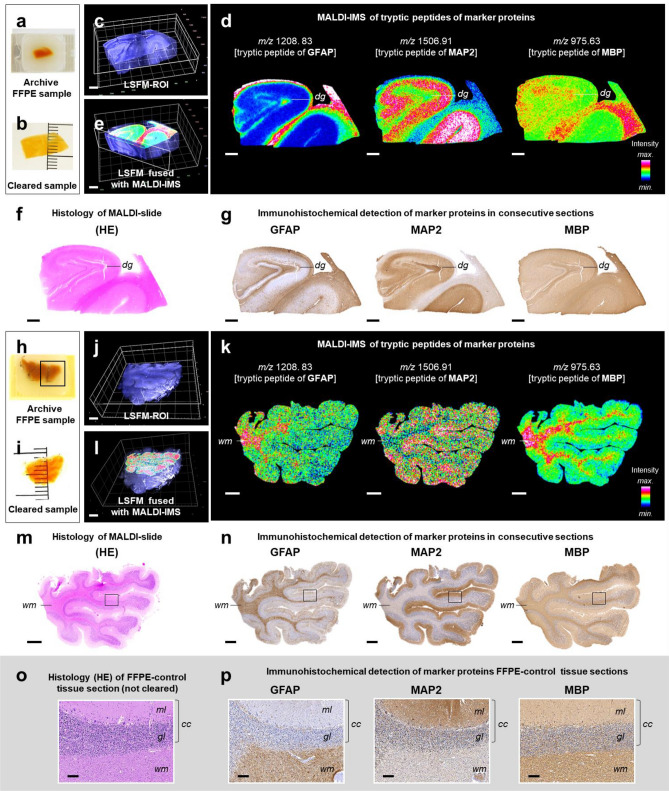
LSFM-guided acquisition of MALDI-MS images in paraffin sections of optically cleared tissue samples prepared from archived human formalin-fixed and paraffin-embedded (FFPE) brain specimen. (**a**–**g**) Dentate gyrus (hippocampus). (**h**–**n**) Cerebellum. (**a**,**h**) FFPE human brain samples. (**b**,**i**) Transparent tissue samples after chemical clearing (3DISCO). (**c**,**j**) 3D LSFM images of the cleared tissue samples (detection of tissue autofluorescence). The section planes of the tissue ROIs selected for subsequent MALDI-MSI analysis are indicated. (**d**,**k**) Representative MALDI-MS images of trypsin-treated paraffin-sections prepared from the tissue ROIs excised from the cleared samples after LSFM. Distinct brain structures are indicated for orientation: Dentate gyrus (*dg*); white matter (*wm*). The presented MALDI-MS images show the spatial distribution and abundance of detected tryptic peptides (confirmed by LC–MS/MS analysis) of well-established marker proteins of different neuronal tissue components with specific histomorphological distribution patterns in the brain [compare to (**f**,**g**,**m**,**n**)]: tryptic peptide GVDAQGTLSK + H^+^ (*m/z* 975.63) corresponds to myelin basic protein (MBP), a marker of myelinating glia, highly abundant in the white matter. Tryptic peptide TTAAGGESALAPSVFK + H^+^ (*m/z* 1,506.91) corresponds to microtubule-associated protein 2 (MAP2), a neuronal differentiation marker, with a particularly distinctive abundance pattern in the molecular layer of the cerebellar cortex (compare to n, p). Tryptic peptide EAASYQEALAR + H^+^ (*m/z* 1,208.83) corresponds to glial fibrillary acidic protein (GFAP), an astrocyte-marker. (**e**,**l**) Fused image of the 3D-LSFM reconstruction of the cleared brain samples (**c**,**j**) and MALDI-MS images of MBP (**l**, *m/z* 975.63), respectively of MAP2 (**e**, *m/z* 1506.91), acquired in paraffin sections of the excised ROIs. (**f**,**m**) HE-stained sections, prepared after MALDI-MSI and matrix removal. Note the structured histo-architecture in the examined brain sections with their different, morphologically clearly distinguishable tissue compartments. (**g**–**n**) Immunohistochemical detection of the marker proteins GFAP, MAP2, and MBP in consecutive sections. Diaminobenzidine (DAB, brown color) was uses as chromogen, and hemalaun (blue color) as nuclear counterstain. Note the concordance of the immunohistochemically detected marker protein abundance patterns with the MALDI-MSI intensity distribution patterns of the corresponding tryptic peptides. Black rectangles in m and n indicate tissue areas containing cerebellar cortex (*cc*) and adjacent white matter (*wm*), corresponding to the tissue regions shown in (**o**) and (**p**). Histology (**o**) and immunohistochemistry (**p**) control sections of formalin-fixed, paraffin-embedded (not cleared) human brain tissue (cerebellum). The molecular (*ml*) and granular (*gl*) layers of the cerebral cortex (*cc*) and the cerebellar white matter (*wm*) are indicated. Note the concordance of the immunohistochemically detected marker protein abundance patterns in sections of control FFPE-cerebellar tissue (**p**) and sections of FFPE-cerebellar tissue that underwent 3DISCO clearing and re-embedding in paraffin (**n**). Bars = 1 mm (**a**–**m**) and 100 µm in (**o**,**p**).

In Experiment No. 2, two samples of FFPE human brain tissue were examined, that had been stored in a pathology archive for 36 and 28 years, respectively. The paraffin-embedded tissue was deparaffinized, optically cleared following the same protocol as in Experiment No. 1 (modified 3DISCO^45^) and examined by LSFM. Detection of the autofluorescence of the tissue samples by LSFM at two wave length bands (585/40 nm and 690/50 nm) was absolutely sufficient to reconstruct accurate, morphologically detailed 3D-images of the examined specimen, discriminating all relevant anatomically and functionally discernable brain tissue compartments in the examined specimen, including gray and white matter, extra- and intra axial blood vessels, meninges, different cerebellar and cerebral cortical layers, and distinct neuron bands (Fig. 2c,j). For subsequent MALDI-MSI analysis, tissue regions of interest were defined by LSFM, including coronal sections of the cerebral- and of the cerebellar cortex. Using anatomical landmarks for orientation, the respective ROIs could easily be excised from the cleared tissue samples. Maintaining their spatial orientation, the excised tissue samples were then re-embedded in paraffin and sectioned until the position and orientation of the section plane corresponded to the previously defined ROI. MALDI-MSI was performed on trypsin pre-digested paraffin sections of the re-embedded tissue, followed by matrix removal and H&E staining of the identical section. For conformational analyses, additional tissue sections were subsequently cut from the same paraffin blocks as the MALDI-MSI sections and processed for proteomic LC–MS/MS analysis, or immunohistochemistry, respectively.

### MALDI-MSI and LC–MS/MS can identify spatial abundance-patterns of proteins and tryptic peptides in paraffin-sections prepared from differentially fixed, optically cleared brain tissue samples

Using the described standard MALDI-TOF-MSI analysis settings, mass spectra (mass range 2.5–25 kDa) of molecular ions suspected to represent proteins could be acquired in the three examined paraffin sections of PaxGene-fixed, optically cleared murine brain tissue. Representative MS-spectra are shown in the supplemental data (Supplemental Fig. 3). Proteins were identified by matching of MALDI-MSI data with the results of the confirmatory LC–MS/MS proteomic profiling experiment. Automatic alignment of MALDI-MSI images of the LC–MS/MS-confirmed proteins and HE-section images was performed using FlexImaging software with external position. Figure 1f,g show representative MALDI-MS images of six annotated proteins with specific spatial abundance- and intensity-patterns reflecting different morphological tissue compartments of the brain. An exemplary illustration of the spatial position of 2D-MALDI-MSI images acquired in the selected ROI-section planes within the 3D-LSFM reconstruction of the cleared mouse brain is provided in Fig. 1e and in Supplemental video 1. In the trypsin-treated paraffin-sections of 3DISCO-cleared human FFPE-brain tissue samples, MALDI-MSI spectra were captured within a mass range between 800 to 4,000 Da. By comparison with LC–MS/MS analysis data acquired in the same sample material, MALDI-MSI-peaks referring to tryptic peptide fragments were identified. Figure 2d,k show representative MALDI-MS images of LC–MS/MS-confirmed tryptic peptides of three established neuronal tissue marker proteins (GFAP, MBP, and MAP2). The MALDI-MS images of these markers match precisely with distinct morphological tissue compartments of the brain (Fig. 2f,m), as well as with their known, specific immunohistological distribution-patterns (Fig. 2g,n,p). The specificity of the immunohistochemical marker-detection in paraffin sections of 3DISCO-cleared brain tissue was confirmed in control sections of (uncleared) FFPE-sections of the same brain tissue samples (Fig. 2o,p). The positions of two MALDI-MSI image planes within the 3D-LSFM reconstructions of the cleared brain samples are exemplarily illustrated in Fig. 2e,l.

## Discussion

In the present study, we used a straight-forward experimental design to provide the *proof-of-principle* for the meaningful combinability of MALDI-MSI and LSFM of optically cleared tissue samples. To demonstrate the broad applicability, the potential, and the benefits of the coalition of these two analysis methods, we examined representative sample types, frequently investigated in LSFM and MALDI-MSI studies, murine brain, and archived human FFPE brain tissue. Widely used, standardized processing- and analysis protocols for optical tissue clearing^45^, paraffin-embedding, and MALDI-MSI of proteins were applied, with unsophisticated, well established, and broadly available materials, analysis-instruments, and -settings. The mouse brain examined in Experiment No. 1 was derived from a previous study that investigated the spatial distribution of fluorescent-labeled leptin, an appetite-regulating 16 kDa hormone that is postprandially released from adipocytes^46^. The mouse brain had been fixed in PaxGene, a formalin-free, commercially available fixation solution, which is recently being used increasingly to avoid the health risks associated with the use of formaldehyde solution-based fixatives, as well as the formaldehyde-induced cross linking of proteins, which is unfavorable for MALDI-MSI^42^. The human brain tissue samples examined in Experiment No. 2, on the other hand, were FFPE specimen, representing the worldwide standard of tissue preservation of the last 100 years. The FFPE samples analyzed in the present study had actually been archived for more than three decades, i.e., long before MALDI-MSI and LSFM had been developed.

For optical clearing of the tissue samples, the established 3DISCO method^45^ was chosen, since this widely used, quick and simple clearing technique produces dimensionally stable, resilient samples that can be precisely sectioned and display only a low degree of shrinkage. Other commonly used clearing techniques, such as CLARITY, also produce excellent tissue clearing results, but are often more complex and time consuming and may require specific technical instrumentation^4^. Since other clearing protocols were not tested in the present study, a comparative assessment of their suitability for subsequent MALDI-MSI analyses cannot be made here.

Expectedly, LSFM of the mouse brain was unproblematic, providing a high-quality 3D reconstruction of the topology of the fluorescent-labeled anatomical structures in the murine brain^46^. For LSFM-based 3D imaging of the unlabeled, deparaffinized and cleared human brain specimen, the natural autofluorescence of the tissue sample was detected in two bandpass filter emission channels at 585/40 nm and 690/50 nm. Detection of autofluorescence proved to provide all necessary morphological tissue information sufficient to reconstruct precise 3D images with remarkable resolution and detail detectability of the different discernable anatomical structures and tissue compartments within the samples. The possibility to receive precise LSFM images of the tissue morphology of archived, belatedly cleared FFPE tissue samples by detection of autofluorescence is particularly significant. It allows to retrieve biologically relevant morphological information of the tissue, that is comparable to that of classical histological stains (of 2D sections), but without the necessity of previous in vivo, or separate postmortal fluorescent labeling or staining procedures of the examined sample. Label-free autofluorescence microscopy for functional and morphological tissue analyses is currently experiencing a true renaissance in life sciences, also driven by the rapidly developing techniques for image registration and mapping^22^ and the capabilities of deep machine learning in detection, recognition and analysis of histo(morpho)logical patterns^48^. For correlation of MSI data with histomorphology, previous studies have already demonstrated the beneficial combination of autofluorescence microscopy with MALDI-MSI in multimodal analyses of (2D)-tissue sections^21,22^. Here, we used autofluorescence- and label-based LSFM, to define different regions of interest (ROIs) in optically cleared, 3-dimensional brain tissue samples for subsequent examination by MALDI-MSI. The examined ROIs within the murine brain (cerebellum with brainstem, diencephalon at the level of the hypothalamus, cerebral cortex/striatum) and human brain (cerebral and cerebellar cortex) were chosen according to the localization of functionally relevant anatomical brain structures and due to their heterogenous tissue composition of white- and grey matter-compartments. Using identifiable anatomical landmarks, and a self-constructed sample positioning and sectioning device, the selected ROIs could conveniently be excised from the cleared tissue samples. For large samples, such as complete mouse brains, with abundant distinctive anatomical details, the use of a positioning and sectioning device is dispensable. However, experimenters might find such a device helpful to achieve precisely oriented section planes when cutting small-sized tissue samples, or cleared specimens with less distinguishable morphological details.

Maintaining their orientation, the excised samples were subsequently re-embedded in paraffin. Sectioning, mounting on MALDI-MSI slides, tryptic digestion of formalin-fixed human brain sections, coating of sections with matrix, and subsequent MALDI-MSI were performed following routine procedures without any technical difficulties. The applied trypsin digestion protocol has been successfully applied in several previous MALDI-MSI studies investigating formalin-fixed tissue sections^16^. To provide the proof for the principal feasibility of MALDI-MSI on paraffin-embedded sections of optically cleared tissue samples, we performed our analyses using a widely used, fast operating Bruker Ultraflex III MALDI-TOF/TOF mass spectrometer.

Proteins, or (tryptic) peptides corresponding to *m/z* values detected by MALDI-MSI were identified by parallel proteomic LC–MS/MS analyses of the same 3DISCO-cleared and paraffin-embedded samples of murine and human brain tissue. Using LC–MS/MS as an independent, methodologically different proteomic analysis approach thus confirmed the successful application of MALDI-MSI protein- and peptide analyses on 3DISCO cleared brain tissue samples.

In the present study, the successful implementation of MALDI-MSI protein/peptide analyses of 3DISCO-cleared brain tissue samples was also confirmed by comparison of MALDI-MS images with the HE-histology of the identical sections, and by additional immunohistochemical analyses of established marker proteins. The MALDI-MSI distribution patterns of several identified masses displayed an accurate, specific overlap with distinct histomorphological structures present in the examined section^16^, discriminating e.g., white and grey matter, ventricles, functionally different compartments such as the thalamus, different layers of the cerebellar cortex, and the brain stem. In sections of 3DISCO-cleared human FFPE brain tissue specimen, the specific, well-known immunohistomorphological distribution patterns of the marker proteins GFAP, MBP, and MAP2 were well retained, as compared to control sections taken from the same brain tissue samples prior to the clearing procedure, and also precisely matched with the distribution patterns of tryptic peptides of these markers detected by MALDI-MSI. These findings confirm the peptide/protein identification results in 3DISCO-cleared FFPE brain samples by MALDI-MSI and LC–MS/MS, and show that the clearing procedure did not induce a significant lateral diffusion of the examined proteins altering their localization in the tissue.

Additionally, in the paraffin sections of the PaxGene-fixed mouse brain, the detected MALDI-MSI abundance patterns of the identified protein GNG3 corresponded well to its immunohistochemical distribution patterns detected in a previous study^47^.

These results thus provide the *proof-of-principle* that MALDI-MSI protein- and peptide-analyses can be performed on 3DISCO-cleared (brain) tissue samples.

Several factors influence the mass detection range and number of identified masses by means of MALDI-MSI, including, the type of tissue, the processing of the samples, and the type of used mass spectrometer^16,19^. During processing of tissue samples for 3DISCO-clearing and paraffin-embedding, the tissue is repeatedly exposed to fixatives, several solvents, including water, ethanol, iso-propanol, xylene, elevated temperatures of up to 65 °C, paraffin, and diverse organic clearing chemicals, such as tetrahydrofuran, dichloromethane, and benzyl alcohol-benzyl benzoate. These processing steps will inevitably alter, attenuate, or remove a significant portion of e.g., lipids and other soluble small molecules from the tissue sample, and might also limit the number of low abundant mass species that can be detected by subsequent MALDI-MSI. Therefore, MALDI-MSI analyses of paraffin sections of 3DISCO-cleared tissue samples appear to be largely confined to peptides- and proteins within the applied MS-mass detection ranges.

Additional studies are necessary to define the single effects of different fixation methods, embedding media, clearing protocols, MALDI-matrices, and particular analysis settings on the range of different classes of analytes that can be examined, and on the number of masses that can be detected by MALDI-MSI using sections derived from optically cleared tissue samples, as compared to e.g., fresh-frozen tissue samples. Future studies will also address the benefits of other MS instrumentations and IMS analysis techniques such as e.g. nanoparticle-based surface assisted laser desorption ionization (LDI)-MS^49^, for the analysis of optically cleared tissue samples.

The combinability of LSFM and MALDI-MSI indicates the potential of using LSFM as a fast scouting method to define specific regions of interest within a 3D tissue sample for subsequent 2D MALDI-MSI analysis of proteins/peptides. In the past, direct 3D MALDI-imaging approaches that aim to visualize and analyze the three-dimensional spatial tissue distribution of diverse analytes identified by MALDI-IMS have been reported^27,50–52^. However, these approaches are generally technically demanding and currently limited to analysis of comparably small samples. In contrast, integration of MALDI-MSI as an additional research modality into the analytical pipeline of an LSFM experiment would allow for multi-level molecular imaging and 2D histology of precisely defined section levels that can be integrated with the 3D histo-architecture of the identical sample. In the present study, illustrative examples for an integration of both imaging modalities into a common 3D image are provided (Figs. 1e, 2e,l, Supplemental video 1). For this, a basic, simple image fusion approach was applied, using distinct anatomical landmarks to align the positions and the lateral dimensions of the selected (2D-) MALDI-MSI images with the (3D-) LSFM reconstructions of the corresponding samples. However, due to the dimensional complexity of the tissue, the lack of direct, unambiguous spatial reference points, and the nonlinear tissue deformation occurring during the different sectioning- and re-embedding processes, this image fusion approach is only capable to achieve an approximate spatial correlation of LSFM-data and MALDI-IMS images. In homogenous tissue samples without unambiguously identifiable anatomical or (histo)morphological landmarks, the achievable (3D-) reposition accuracy of both imaging modalities might be more restricted, probably not even reaching the lateral resolution of MALDI-MSI image. To take full benefit from the combination of LSFM with additional imaging modalities, such as and MALDI-MSI, histology, or immunohistochemistry, future studies might apply advanced methods of computational digital image coregistration, alignment and fusion of 3D- with 2D-image data^27^, such as those recently described by Abdelmoula et al*.* for non-linear alignment of MALDI-IMS images with 3D-MRI-image-datasets^24^. Also the application of external fiducial masks, i.e., external (physical) position markers which are added to the cleared tissue, and which can unambiguously be identified in the (virtual) 3D-LSFM sample reconstruction as well as in subsequently generated histological sections and MALDI-IMS images, might be useful for the precise excision of LSFM-defined ROI section planes from the cleared 3D tissue sample, as well as for the accurate fusion (“back-matching”) of 2D-MALDI-MSI images with the corresponding 3D-LSFM data. Such external fiducial masks have for example previously successfully been applied for the correlation of mass spectrometry imaging and confocal Raman microscopy^23^ or laser scanning confocal microscopy^26^ in studies of three-dimensional cell cultures.

## Conclusions

Integration of MALDI-MSI with 3D-LSFM of optically cleared tissue samples will be of great value in studies where MALDI-MSI is to be combined with qualitative and quantitative morphological analyses of complex three-dimensional tissue structures, such as neuron interactions in the central nervous system, spatial vascularization patterns, or branching designs of ductular structures in different animal and human tissues during development, health and disease. In this context, the examination of FFPE-samples by combined LSFM-MALDI-IMS approaches is particularly advantageous. It enables retrospective analyses of archived FFPE specimen, which constitute the vast majority of the stocks of pathology- and biobank archives that hold an immeasurably valuable reserve of well-defined samples representing the most diverse physiological and pathological conditions.

## Methods

### Tissue samples

The brain sample examined in Experiment No. 1 was derived from an adult, male C57/BL6J mouse that was examined in a previous study investigating the distribution of leptin, an adipose tissue derived peptide hormone in the brain. A detailed description of the applied methods is provided in the original publication^46^. Briefly, the mouse was i.p.-injected with recombinant mouse leptin (5 mg/kg), labeled with a fluorescent dye (IRDye, CW800, #929-71012, LICOR, Germany), and i.v.-injected with 250 µg fluorescent lectin-647 (Cat# L32451, ThermoFisher Scientific Inc., USA) to visualize blood vessels. The animal was killed by cervical dislocation, the brain was removed and fixed in PaxGene (PreAnalytiX, Switzerland) according to the manufacturer’s recommendations and then optically cleared as described below.

The archived human FFPE brain tissue samples examined in Experiment No. 2 had been stored in a pathology archive for 36 and 28 years, respectively, prior to examination in the present study.

### Chemical clearing of tissue samples and LSFM

Chemical clearing of tissue samples was performed, according to a modified version of the 3DISCO protocol^45^. The PaxGene-fixed mouse brain, respectively the de-paraffinized and rehydrated FFPE-tissue samples of human brain tissue were processed through series of clearing solutions: 50% tetrahydrofuran (THF, 1 h), 50% THF (12 h), 70% THF (8 h), 80% THF (12 h), 100% THF (12 h), 100% THF (1 h), 100% dichloromethane (DCM, 30 min until tissue sample sinks), benzyl alcohol-benzyl benzoate (BABB, 10 min), BABB (20 min), BABB (30 min). The samples were then stored in BABB in the dark for 2 days at 4–8 °C. THF, DCM, and BABB were purchased from Sigma-Aldrich, Germany. Cleared tissues were subsequently examined by LSFM with benzyl alcohol-benzyl benzoate (BABB) as medium, using an UltraMicroscope II (LaVision BioTec GmbH, Germany) equipped with a SuperK EXTREME EXW12 white laser (NTK Photonics, Cologne, Germany) and a 2 × objective lens (Olympus MVPLAPO 2 ×/0.5 NA) combined with an Olympus MVX-10 zoom body (Olympus, Hamburg, Germany). Z-stacks of fluorescence images of 5 µm optical thickness were acquired at 520/40 nm (excitation range) 585/40 nm (emission range) and 640/30 nm (excitation range) 690/50 nm (emission range) for detection of autofluorescence in human brain samples. For LSFM imaging of the mouse brain, a bandpass filter set with an excitation range of 640/30 nm and emission range of 690/50 nm was used for detection of fluorescent lectin-647 signals (visualization of blood vessels) in combination with an additional filter set (excitation: 710/7 nm; emission: 810/90 nm) for detection of fluorescent leptin-CW800 signals. With the used objective lens and the applied analysis settings, also a lateral resolution of approximately 5 µm was achieved. 3D images were computed, using ImSpector Pro^64^ (vers. 5.1.328, LaVision Biotec GmbH, Germany), and arivis Vision4D (vers. 3.0, arivis, Germany) software tools.

### Excision of regions of interest from optically cleared tissue samples and subsequent paraffin-embedding

In the cleared brain tissue samples, ROIs for subsequent MALDI-MSI analysis were defined, using LSFM. In the mouse brain, three coronal (frontal) section planes were specified as ROIs, at the level of the cerebral cortex/striatum, the thalamus, and the cerebellum/brain stem. ROIs from human brain tissue samples included coronal sections of the cerebral and the cerebellar cortex. Following LSFM, the respective tissue-ROIs were excised from the cleared tissue samples, using anatomical landmarks. To facilitate cutting of straight, controlled section planes, a self-constructed device for three-dimensional sample positioning and sectioning was used (Supplemental Fig. 2). Excised tissue ROIs were subsequently submerged in 100% isopropyl alcohol for 2 × 15 min, and then transferred to 100% ethanol for 2 × 15 min, and 70% ethanol for 2 × 15 min, followed by embedding in paraffin, following a standard protocol, using a vacuum infiltration processor (Tissue-Tek VIP 5, Sakura, Japan). Sections of a thickness of 3 µm were cut from the paraffin-blocks, using a Microm HM 355S microtome (HistoServe, Celle, Germany). For MALDI-MSI analysis, sections were mounted on 1:1 (v/v) poly-l-lysine: 0.1% Nonidet P-40 pretreated indium-tin-oxide glass slides (Bruker Daltonics, Bremen, Germany). For subsequent validation of MALDI-MSI data, by proteomic LC–MS/MS analyses and immunohistochemistry (described below), additional (consecutive) sections were cut from the same paraffin blocks.

### MALDI-MSI

In both experiments, a standard sample processing and analysis pipeline was applied for MALDI-MSI of proteins, using a Bruker Ultraflex III MALDI-TOF/TOF MS (Bruker Daltonics).

### MALDI-MSI of intact proteins in paraffin sections of PaxGene-fixed and optically cleared murine brain tissue

Sections of the defined ROIs of the optically cleared and paraffin-embedded mouse brain tissue were dried and adhered by incubating the slide for 1 h at 70 °C. Remaining paraffin was removed with xylene (2 × 3 min) and the slides were washed with 2-propanol (3 min). For tissue rehydration, slides were incubated in ethanol dilutions decreasing from 100%, 90%, 70%, 50% to 30% (1 min, each). Following evaporation of ethanol at 40 °C, 10 g/l sinapinic acid matrix (Sigma-Aldrich) in 60% acetonitril/0.2% trifluoroacetic acid was deposited onto the sections using an ImagePrep automated sprayer (Bruker Daltonics). MALDI-TOF-MSI was carried out in linear positive ion mode over a mass range of *m/z* 2.5–25 kDa. The lateral resolution was set to 50 μm. The Smartbeam-II Nd:YAG (355 nm) laser frequency was set to 200 Hz with 300 shots per spot and a sample rate of 0.50 GS/s. External calibration was performed with Protein Calibration Standard I (Bruker) mixed 1:1 (v/v) with matrix solution and spotted onto the slide.

### MALDI-MSI of digested proteins (tryptic peptides)

Sections prepared from deparaffinized, cleared and re-paraffin-embedded human archive FFPE brain samples were dried and adhered by incubating the slide for 1 h at 70 °C and remaining paraffin was removed with xylene (2 × 5 min). For tissue rehydration, slides were incubated in ethanol dilutions decreasing from 100% (2 × 3 min), 70% (1 min) to 30% (1 min), followed by two washing steps in ultrapure water (2 × 3 min). Due to the initial formalin-fixation of the specimen, the paraffin sections prepared for MALDI-MSI were subjected to a trypsin pre-digestion step prior to coating with MALDI-matrix. Antigen retrieval was performed at 97 °C in 10 mM citric acid (pH 6) for 30 min. Thereafter, cooled slides were washed for 5 min in ultrapure water and subsequently dried, followed by coating with 15 layers of 0.02 µg/µl trypsin gold (Promega, Madison, USA) in ultrapure water at a constant flow rate of 10 µl/min using a SunCollect sprayer (Sunchrom, Friedrichsdorf, Germany). Proteins were digested at 37 °C and 97% humidity for 18 h in a SunDigest Incubation Chamber (Sunchrom). 7 g/l α-cyano-4-hydroxycinnamic acid in 50% acetonitrile/0.2% trifluoroacetic acid were sprayed on the sections using a SunCollect sprayer (Sunchrom). The first three layers were sprayed with increasing flow rates of 10, 20 and 30 µl/min, while 40 µl/min was used for layers four to seven. MALDI-TOF-MSI was carried out in reflector positive ion mode over a mass range of *m/z* 800–4,000 with a lateral resolution of 50 µm. The Smartbeam-II Nd:YAG (355 nm) laser frequency was set to 100 Hz with 200 shots per spot and a sample rate of 2.00 GS/s. External calibration was performed with Peptide Calibration Standard II (Bruker) mixed 1:1 (v/v) with matrix solution and spotted onto the slide.

### Hematoxylin and eosin staining and slide digitalization

Following MALDI-MSI, the matrix was washed off the section surface in 70% ethanol for 4 min. The sections were subsequently stained with hematoxylin–eosin in a HistoCore SPECTRA ST multistainer (Leica, Germany), using the ST Infinity H&E staining system (ref. 3801098, Leica, Germany), coverslipped, examined by light microscopy, and scanned using a digital slide scanner equipped with a 20 × magnification objective (MIRAX DESK, Zeiss, Germany).

### MALDI mass spectrometry imaging data analysis

MALDI-TOF-MSI data was exported from flexImaging (v.4.0, Bruker). Smoothing and peak picking was performed in mMass (v.5.5.0)^53^ using the Savitzky-Golay algorithm (protein spectra: 5 *m/z* window size, 2 cycles; peptide spectra: 0.2 *m/z* window size, 2 cycles) and a S/N of 3 (proteins) or a S/N of 0.5 with a relative intensity threshold of 0.4 (peptides).

### Protein and peptide identification by proteomic LC–MS/MS analysis

Validation of MALDI-MSI data was performed by proteomic LC–MS/MS analyses. For this, multiple, thick (consecutive) sections containing approximately 1 mg tissue were cut from the same paraffin blocks of (optically cleared and re-embedded) human and murine brain tissue samples, the MALDI-MSI sections had previously been cut from (see above). Following dewaxing of the sections in xylene for 4 × 10 min, the deparaffinized tissue was resuspended in Tris-buffered saline and incubated for 1 h at 99 °C for antigen retrieval. After cooling to room temperature urea buffer was added to reach a final concentration of 4.5 M urea in 100 mM Tris/HCl pH 8.5. Tissue was bead milled (Precellys Homogenizer, Bertin Technologies SAS, France) and total protein content was measured by Bradford assay. 10 µg total protein of each sample was proteolysed by modified filter aided sample preparation (FASP) as described^54,55^. LC–MS/MS analysis was performed on a QExactive HF mass spectrometer (ThermoFisher Scientific, Waltham, USA) online coupled to an Ultimate 3000 RSLC nano-HPLC (Dionex, Sunnyvale, USA). Samples were automatically injected and loaded onto the C18 trap column and after 5 min eluted and separated on the C18 analytical column (Acquity UPLC M-Class HSS T3 Column, 1.8 μm, 75 μm × 250 mm; Waters, Germany) by a 90 min non-linear acetonitrile gradient at a flow rate of 250 nl/min. MS spectra were recorded at a resolution of 60,000 and after each MS1 cycle, the 10 most abundant peptide ions were selected for fragmentation. Acquired raw data were analyzed using Mascot (Matrix Science, UK; version 2.6.2). Mascot was set up to search the SwissProt database either specifying *Homo sapiens* or *Mus musculus* as species and assuming the digestion enzyme trypsin, allowing for one missed cleavage site. Mascot was searched with a fragment ion mass tolerance of 0.020 Da and a parent ion tolerance of 10 PPM. Carbamidomethyl of cysteine was specified as a fixed modification. Deamidated of asparagine and glutamine and oxidation of methionine were specified as variable modifications. Scaffold (version Scaffold_4.8.2, Proteome Software Inc., USA) was used to validate MS/MS based peptide and protein identifications. Protein and peptide annotation was done by matching of MALDI-MSI to the LC–MS/MS data. Peptide identifications were accepted if they could be established at greater than 91% probability to achieve an FDR less than 1% by the Scaffold Local FDR algorithm. Protein identifications were accepted if they could be established at greater than 95% probability and contained at least one identified peptide. Protein probabilities were assigned by the Protein Prophet algorithm^56^. Proteins that contained similar peptides and could not be differentiated based on MS/MS analysis alone were grouped to satisfy the principles of parsimony. Proteins sharing significant peptide evidence were grouped into clusters.

### Immunohistochemistry

For confirmation of MALDI-MSI results, the spatial abundance patterns of different established nerve tissue markers (myelin basic protein, MBP; microtubule-associated protein 2, MAP2; glial fibrillary acidic protein, GFAP) were analyzed by immunohistochemistry (IHC), using specific antibodies (monoclonal mouse anti-MAP2-antibody, clone AP18, cat.-No.: MAK 6008, LINARIS Biologische Produkte GmbH, Germany; polyclonal rabbit anti-MBP antibody, cat.-No.: A0623, DAKO/Agilent, USA; polyclonal rabbit anti-GFAP antibody, cat-No.: Z0334, DAKO/Agilent, USA). For detection of immunoreactivity, the ImmPRESS HRP Universal (Horse Anti-Mouse/Rabbit IgG) PLUS Polymer Kit—Peroxidase (cat.-No.: MP-7800, Vector Labs. Ltd., UK) was applied. IHC analyses were performed on consecutive sections of the identical samples of optically cleared and re-embedded human brain tissue previously analyzed by MALDI-MSI. All IHC analyses included appropriate negative controls (omission of the first antibody). Sections of FFPE human cerebellar tissue (not cleared) served as positive control.

### Alignment of MALDI-MSI images with HE-images of the same sections and fusion with 3D-LSFM reconstructions

Automatic alignment of MALDI-MSI imaging data and H&E-section images was performed, using the Co-Register Image dialog in FlexImaging software (Bruker Daltonik, Bremen) (Ref.: PMID: 22011652). For three-dimensional visualization, representative MALDI-MSI images of selected masses were merged into the 3D-LSFM image z-stacks at the positions of the respective ROIs (Figs. 1e, 2e,l, Supplemental Video 1). Anatomical landmarks were used to align the positions and lateral dimensions of the (2D) MALDI-MSI planes with the 3D-LSFM reconstructions of the corresponding samples, using the Volume Fusion mode of the arivis Vision4D (vers. 3.0, arivis, Germany) software.

### Ethical statement

The mouse brain examined in Experiment No. 1 was derived from a previously published study^46^. The animal experiments in this study were approved by the institutional ethics committee of the State of Bavaria and performed in accordance with the relevant guidelines and regulations and with permission of the local authorities (ROB-55.2Vet-2532.Vet_02-16-99).

The FFPE samples of human brain tissue examined in Experiment No. 2 were derived from two routine autopsy cases performed in 1982, and in 1990, and had been stored in the pathology archive of the Institute for Pathology of the Technische Universität München ever since. The use of these archived samples for scientific experiments was approved by the ethical committee of the Technische Universität München and performed in accordance with the Declaration of Helsinki and national legal regulations.

## Supplementary information

Supplementary Information.

Supplementary Video.

## Data Availability

The datasets generated during and/or analyzed during the current study are available from the corresponding author on reasonable request.
