# Supplementary material for: Light sheet fluorescence microscopy guided MALDI-imaging mass spectrometry of cleared tissue samples
Source: Sci Rep. 2020 Sep 2;10:14461. doi: 10.1038/s41598-020-71465-1 (PMC7468256; doi:10.1038/s41598-020-71465-1)
Supplement: Supplementary file 1 — Supplementary Information. [file 41598_2020_71465_MOESM1_ESM.docx]

**Light sheet fluorescence microscopy guided MALDI-imaging mass spectrometry of cleared tissue samples**

Andreas Blutke^1^, Na Sun^1^, Zhihao Xu^1^, Achim Buck^1^, Luke Harrison^2,3,4,5^, Sonja C. Schriever^2,3,4^, Paul T. Pfluger^2,3,4^, David Wiles^6^, Thomas Kunzke^1^, Katharina Huber^1^, Jürgen Schlegel^7^, Michaela Aichler^1^, Annette Feuchtinger^1^*, Kaspar Matiasek^8^, Stefanie M. Hauck^9^, and Axel Walch^1^

^1^Research Unit Analytical Pathology, Helmholtz Zentrum München, 8576 Neuherberg, Germany

^2^Research Unit Neurobiology of Diabetes, Helmholtz Zentrum München, 85764 Neuherberg, Germany

^3^Institute for Diabetes and Obesity, Helmholtz Zentrum München, 85764 Neuherberg, Germany

^4^German Center for Diabetes Research (DZD), 85764 Neuherberg, Germany

^5^Division of Metabolic Diseases, Technische Universität München, 80333 Munich, Germany

^6^arivis AG, 80636 München, Germany

^7^Institute for Pathology, Department of Neuropathology, Technische Universität München, 80333 Munich, Germany.

^8^Institute for Veterinary Pathology at the Centre for Clinical Veterinary Medicine, Ludwig-Maximilians-Universität München, 80539 Munich, Germany

^9^Research Unit for Protein Science, Helmholtz Zentrum München, 85764 Neuherberg, Germany

*Corresponding author. Email: annette.feuchtinger@helmholtz-muenchen.de

**Supplemental data**

**Supplemental Figure 1.** **Schematic experimental design and work-flow of Experiment N°1 and Experiment N°2.**

**
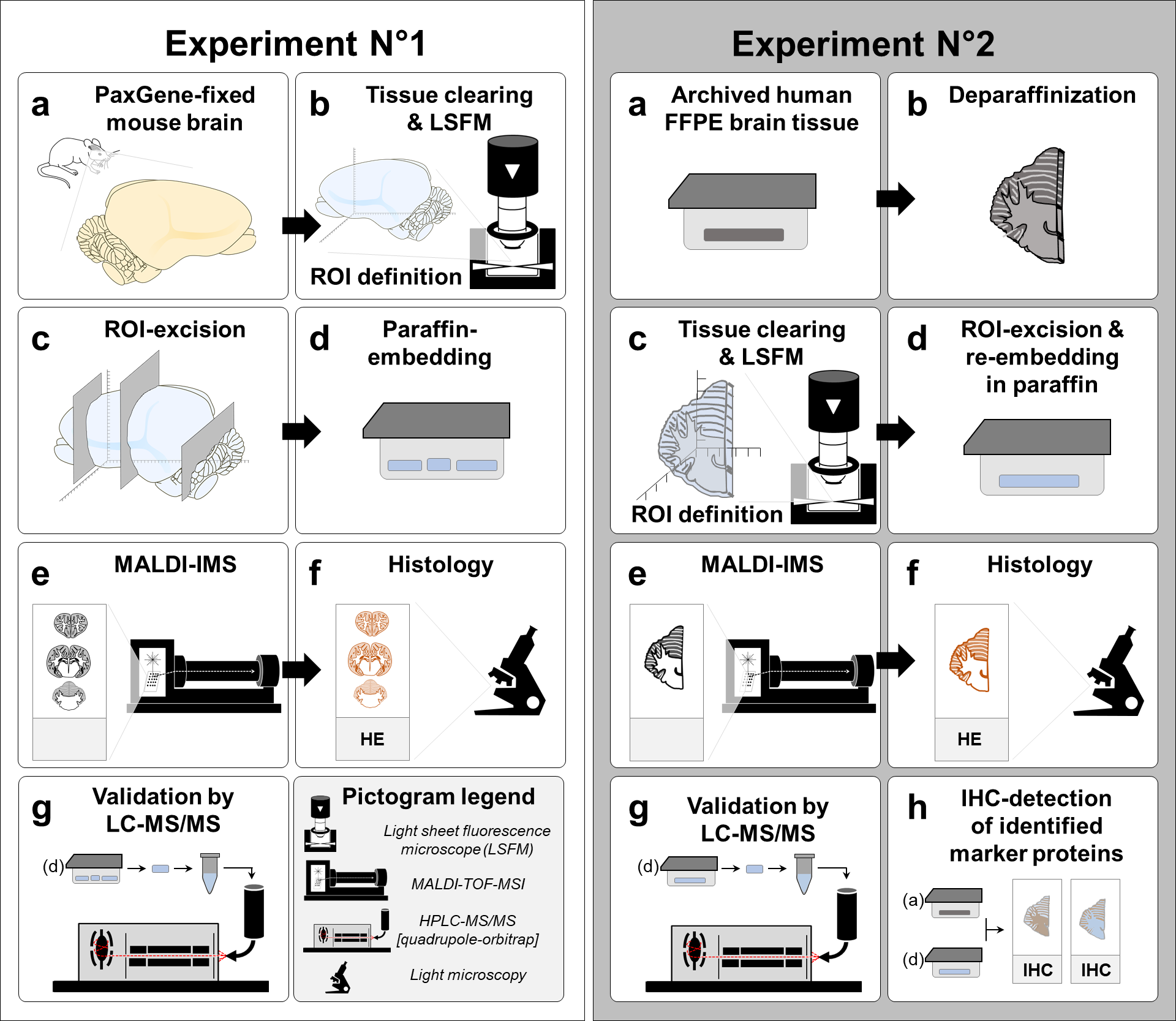
**

**Supplemental Figure 1.** **Schematic experimental designs and work-flows.** To demonstrate the feasibility of MALDI-MSI on paraffin sections of optically cleared tissue samples, two experiments were performed. In **Experiment N°1**, a freshly dissected mouse brain was fixed in formaldehyde-free fixation solution (PaxGene) (**a**), optically cleared (3DISCO) and subjected to light sheet fluorescence microscopy (LSFM) (**b**). Regions of interest (ROIs) defined by LSFM were dissected from the cleared tissue (**c**), and embedded in paraffin (**d**). Paraffin sections were subjected to MALDI-MSI (**e**) and subsequent HE-staining (**f**). For validation of MALDI-MSI data, proteomic LC-MS/MS analysis was performed on additional samples of paraffin-embedded mouse brain tissue (**g**). For **Experiment N°2**, FFPE human brain tissue (archive material, **a**) was deparaffinized, (**b**) optically cleared (3DISCO) and subjected to LSFM (**c**). Regions of interest were defined by LSFM and dissected from the cleared tissue, and re-embedded in paraffin (**d**). Paraffin sections of the re-embedded tissue were subjected to MALDI-MSI (**e**) and subsequent HE-staining (**f**). For validation of MALDI-MSI data, proteomic LC-MS/MS analysis was performed on additional samples of paraffin-embedded human brain tissue (**g**) and the spatial abundance patterns of MALDI-MSI detected tryptic peptides of different established nerve tissue marker proteins were confirmed by immunohistochemistry (**h**).

**Supplemental Figure 2**

**
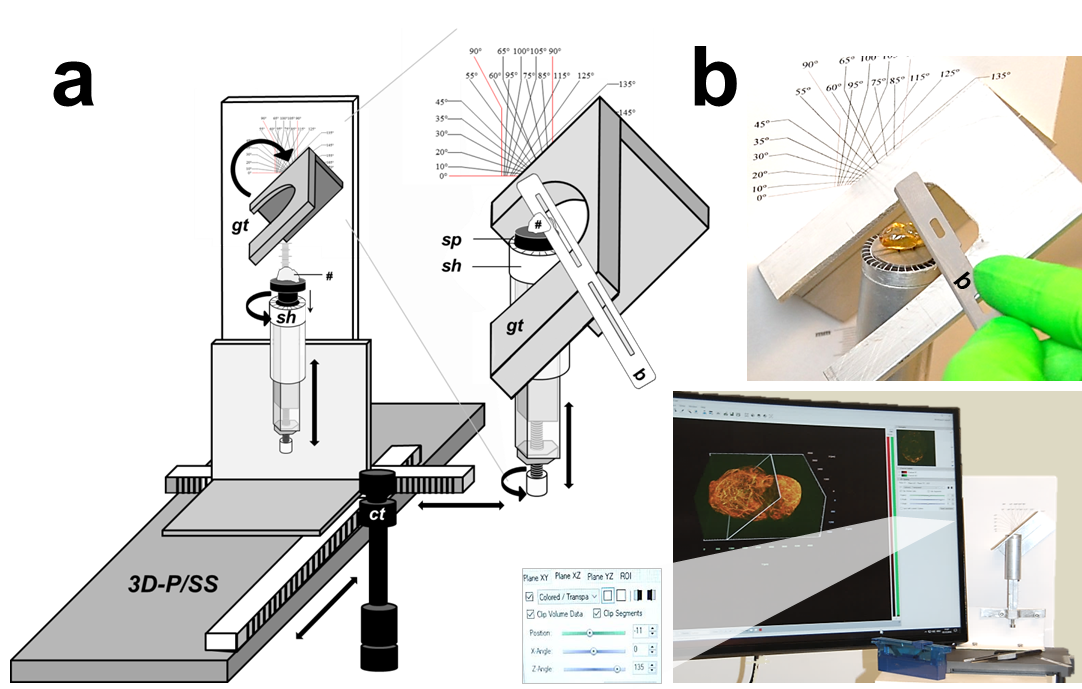
**

**Supplemental Figure 2. Excision of a LSFM-determined ROI from the cleared tissue sample.**

Within the cleared tissue, ROI(s) for subsequent MALDI-MSI are defined by LSFM and subsequently excised from the sample. For reasonably sized organ samples, such as complete mouse brains, with abundant distinctive anatomical details, the use of a positioning and sectioning device is dispensable and sections can be straightforwardly excised by hand, with adequate precision. However, for cutting of small-sized tissue samples, or for preparation of oblique sections through cleared samples with indistinct, uniform external shapes, the aid of a three-dimensional sample positioning device (**3D-P/SS**) with a blade guiding track (***gt***), is advantageous. Here, a self-constructed positioning and cutting-device, built from a microscope cross-table (***ct***) and few standard hardware store components is shown. **a**: Construction scheme. **b:** Gross image and detail enlargement. For accurate positioning of the sample (#) and the section plane(s), the x-, y-, and z-position of the sample holder (***sh***) can be precisely be adjusted, the sample-plate (***sp***) and the blade guiding track can be rotated by 360°. To precisely replicate the orientation of LSFM optical section plane(s), the sample plate with the tissue sample (***#***) is removed from the sample chamber of the LSFM and directly transferred to the sample plate holder of the 3D-positioning/cutting device. The sample-positioning data and the section plane angle(s) of the LSFM-viewer software are correspondingly transferred to the gadget, and the cleared tissue sample is sectioned, using a microtome blade (***b***). Care should be taken to place the physical section plane through the cleared tissue sample approximately 0.5 mm parallel to the optical LSFM-section plane, to avoid cutting directly through the structure of interest. In the present example, the positioning of a cleared mouse brain for preparation of an 135° oblique frontal section through the cerebrum and the diencephalon is shown (the presented example intends to illustrate the sectioning process, it does not show the same ROI-section plane orientation examined in the MALDI-MSI experiment). Note that the described method of tissue-sectioning works well only for solid, shape-retaining optically cleared tissue samples, but not for soft/gelatinous samples.

**Supplemental Figure 3**

**
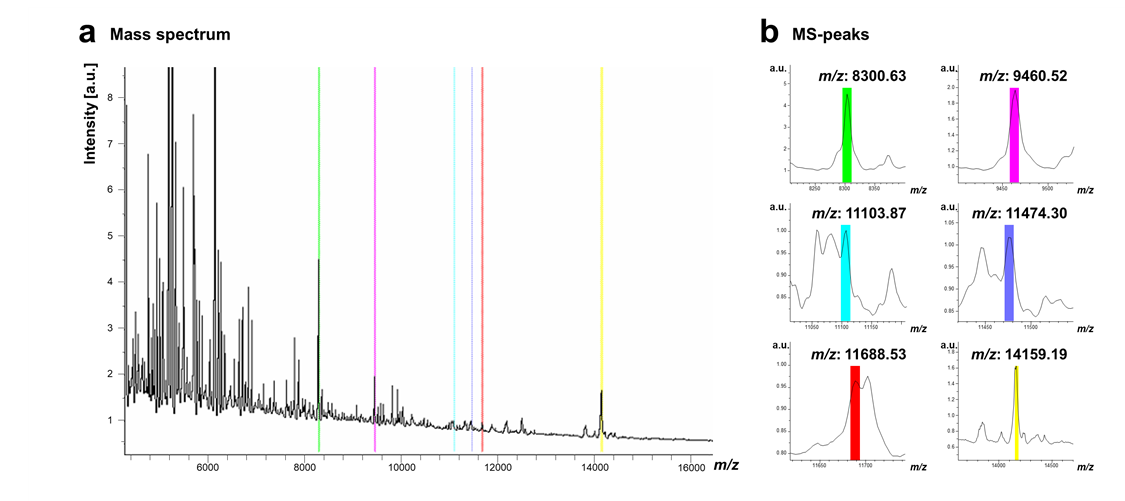
**

| **c**  **Protein** | **Protein accession numbers** | **Protein molecular weight (Da)** | **Protein identification probability** | **Exclusive unique peptide count** | **Sequence coverage (%)** | **Corresponding MALDI-MSI Peak (*m/z*)** |
| --- | --- | --- | --- | --- | --- | --- |
| GNG3 | P63216\|GBG3_MOUSE | 8304.90 | 100.00% | 2 | 60.00% | 8300.63 |
| RPS27 | Q6ZWU9\|RS27_MOUSE | 9460.90 | 100.00% | 2 | 28.60% | 9460.52 |
| MRPS36 | Q9CQX8\|RT36_MOUSE | 11101.10 | 100.00% | 4 | 57.80% | 11103.87 |
| RPLP1 | P47955\|RLA1_MOUSE | 11475.00 | 100.00% | 2 | 51.80% | 11474.30 |
| NDUFB3 | Q9CQZ6\|NDUB3_MOUSE | 11692.30 | 99.10% | 2 | 14.40% | 11688.53 |
| SCOC | Q78YZ6\|SCOC_MOUSE | 14155.00 | 100.00% | 2 | 35.20% | 14159.19 |

**Supplemental Figure 3. MS-spectra of MALDI-IMS and identified proteins validated by LC-MS/MS analysis in experiment N°1 (PaxGene-fixed, 3DISCO-cleared, paraffin-embedded mouse brain tissue).** **a**: MALDI-mass spectrum from 4-16 kDa. MS-protein peaks of six identified masses are highlighted in different colors. **b**: Detail enlargements of these mass spectra peaks with corresponding *m/z* values. The six featured mass spectra correspond to ions of the same LC-MS/MS-confirmed proteins shown in Figure 1. *m/z*: 8300.63: GNG3 (guanine nucleotide-binding protein subunit gamma-3); *m/z*: 9460.52: RPS27 (40S ribosomal protein S27); *m/z*: 11103.87: MRPS36 (28S ribosomal protein S36, mitochondrial); *m/z*: 11474.30: RPLP1 (60S acidic ribosomal protein P1); *m/z*: 11688.53: NDUFB3 (NADH dehydrogenase [ubiquinone] 1 beta subcomplex subunit 3); *m/z*: 14159.19: SCOC (Short coiled-coil protein). Relevant parameters of the LC-MS/MS protein identification are provided in table c.

**Supplemental Figure 4**

**a**

**GFAP (*m/z* 1208.83, EAASYQEALAR + H^+^)**


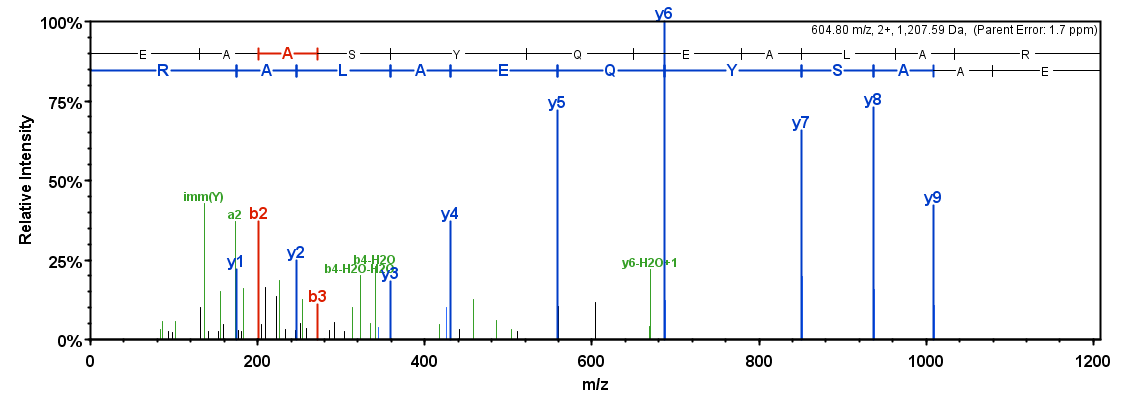


**MAP2 (*m/z* 1506.91, TTAAGGESALAPSVFK + H^+^)**

**
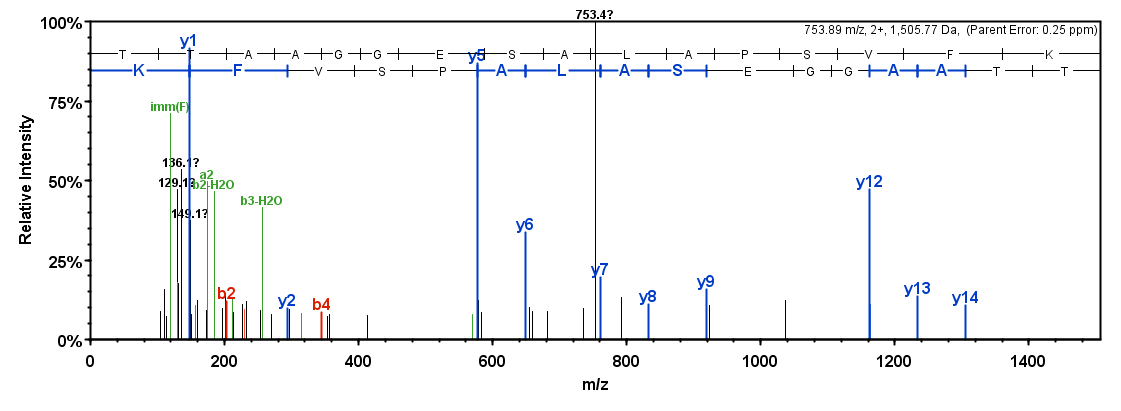
**

**MBP (*m/z* 975.63, GVDAQGTLSK + H^+^)**

**
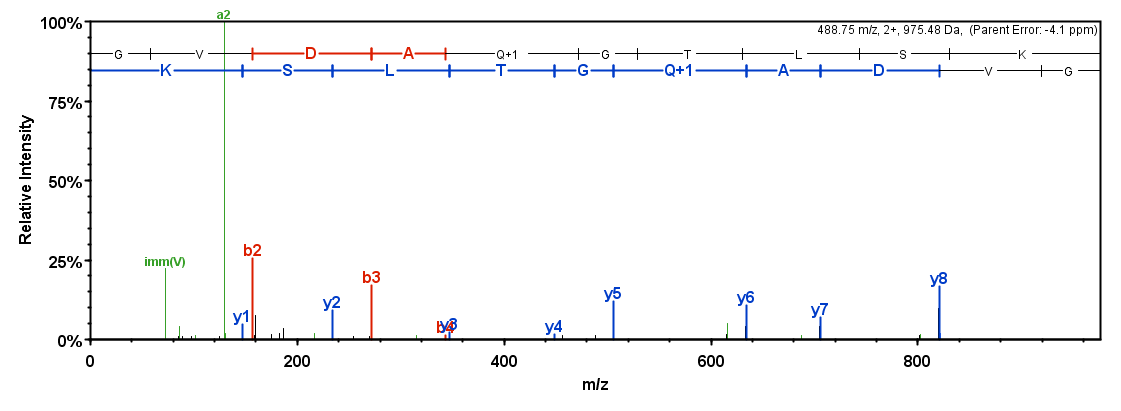
**

**b**

| Protein | Protein accession numbers | Protein molecular weight (Da) | Protein identification probability | Unique peptide count | Peptide sequence | Peptide identification probability | Calculated +1H Peptide Mass |
| --- | --- | --- | --- | --- | --- | --- | --- |
| GFAP | P14136\|GFAP_HUMAN | 49881.40 | 100.00% | 48 | EAASYQEALAR | 99.70% | 1208.59 |
| MAP2 | P11137\|MTAP2_HUMAN | 199527.90 | 100.00% | 34 | TTAAGGESALAPSVFK | 99.70% | 1506.78 |
| MBP | P02686\|MBP_HUMAN | 33117.70 | 100.00% | 4 | GVDAQGTLSK | 99.70% | 975.5107 |

**Supplementary Figure 4. MS/MS-spectra of tryptic peptides detected by MALDI-IMS and validated by LC-MS/MS analysis in experiment N°2 (FFPE, 3DISCO-cleared, and paraffin-(re)embedded human brain tissue samples).** The MS/MS-spectra shown in (a) correspond to ions of tryptic peptides of the same established neural tissue marker proteins also detected by MALDI-MSI and confirmed by immunohistochemistry (refer to Figure 2). Tryptic peptide EAASYQEALAR+H^+^ (*m/z* 1208.83) corresponds to glial fibrillary acidic protein (GFAP), an astrocyte-marker. Tryptic peptide TTAAGGESALAPSVFK+H^+^ (*m/z* 1506.91) corresponds to microtubule-associated protein 2 (MAP2), a neuronal differentiation marker. Tryptic peptide GVDAQGTLSK+H^+^ (*m/z* 975.63) corresponds to myelin basic protein (MBP), a marker of myelinating glia, highly abundant in the white matter. Relevant parameters of the LC-MS/MS peptide 3 identification are provided in table b.

**Supplemetal Video 1. LSFM guided MALDI-MSI** **and integration of 2D MALDI-MS images with 3D-LSFM reconstructions of organ morphology.** The video shows the 3D reconstruction of a PaxGene-fixed, optically cleared (3DISCO) mouse brain from a previous study^1^. Blood vessels are visualized in gray color (tagged by fluorescent labeled lectin) [0:00-0:06 sec]. The positions and orientations of the section planes of the tissue regions of interest (ROI N°1-3) selected for excision and MALDI-MSI are superimposed with MALDI-MS images subsequently acquired in paraffin sections of the corresponding locations [0:09-0:34 sec], showing the spatial distributions and intensities of guanine nucleotide-binding protein subunit gamma-3 (Gng3, *m/z*: 8300.63). Signal intensities are visualized by different colors; minimal intensities are shown in black-to blue, maximal intensities are indicated by red color (compare to the intensity-color scales shown in Fig. 1 and Fig. 2).

1. Harrison, L., et al., Fluorescent blood-brain barrier tracing shows intact leptin transport in obese mice. Int J Obes (Lond), 2018.
